# Oncological and functional outcome after laryngectomy for laryngeal and hypopharyngeal cancer: a population-based analysis in Germany from 2001 to 2020

**DOI:** 10.1038/s41598-024-58423-x

**Published:** 2024-04-02

**Authors:** Mussab Kouka, Louise Beckmann, Thomas Bitter, Holger Kaftan, Daniel Böger, Jens Büntzel, Andreas Müller, Kerstin Hoffmann, Jiri Podzimek, Klaus Pietschmann, Thomas Ernst, Orlando Guntinas-Lichius

**Affiliations:** 1 Department of Otorhinolaryngology, Jena University Hospital, Am Klinikum 1, 07747 Jena, Germany; 2 Department of Otorhinolaryngology, Helios-Klinikum Erfurt, Erfurt, Germany; 3 Department of Otorhinolaryngology, SRH Zentralklinikum Suhl, Suhl, Germany; 4 Department of Otorhinolaryngology, Suedharzklinikum Nordhausen, Nordhausen, Germany; 5 Department of Otorhinolaryngology, SRH Wald-Klinikum Gera, Gera, Germany; 6 Department of Otorhinolaryngology, Sophien-Hufeland-Klinikum, Weimar, Germany; 7 Department of Otorhinolaryngology, Klinikum Bad Salzungen, Bad Salzungen, Germany; 8 Department of Radiation Oncology, Jena University Hospital, Jena, Germany; 9 University Tumor Center, Jena University Hospital, Jena, Germany

**Keywords:** Cancer epidemiology, Head and neck cancer, Oncology, Cancer, Surgical oncology

## Abstract

Prognostic factors for overall survival (OS), percutaneous endoscopic gastrostomy (PEG) dependency, and long-term speech rehabilitation via voice prosthesis (VP) after laryngectomy for laryngeal or hypopharyngeal cancer were investigated in a retrospective population-based study in Thuringia, Germany. A total of 617 patients (68.7% larynx; hypopharynx; 31.3%; 93.7% men; median age 62 years; 66.0% stage IV) from 2001 to 2020 were included. Kaplan–Meier and Cox multivariable regression analyses were performed. 23.7% of patients received a PEG. 74.7% received a VP. Median OS was 131 months. Independent factors for lower OS were stage IV (compared to stage II; hazard ratio [HR] = 3.455; confidence interval [CI] 1.395–8.556) and laryngectomy for a recurrent disease (HR = 1.550; CI 1.078–2.228). Median time to PEG removal was 7 months. Prior partial surgery before laryngectomy showed a tendency for independent association for later PEG removal (HR = 1.959; CI 0.921–4.167). Postoperative aspiration needing treatment was an independent risk factor (HR = 2.679; CI 1.001–7.167) for later definitive VP removal. Laryngectomy continuously plays an important role in a curative daily routine treatment setting of advanced laryngeal or hypopharyngeal cancer in Germany. Long-term dependency on nutrition via PEG is an important issue, whereas use of VP is a stable long-term measure for voice rehabilitation.

## Introduction

Total laryngectomy (in the following only laryngectomy) is one primary curative treatment option for advanced laryngeal and hypopharyngeal cancer^1,2^. There is an ongoing debate, if definitive radiochemotherapy is the better alternative^3^. When comparing both therapy strategies, it is important to regard both oncological results like overall survival (OS), but also the long-term functional outcome. Especially, voice and swallowing function can be disturbed after all curative, surgical or non-surgical approaches. Laryngectomy is followed by loss of the natural voice. High standard of speech rehabilitation is phonation through a tracheoesophageal puncture (TEP) with a voice prosthesis. It has clearly been shown that a TEP is leading to a significant increase in quality of life^2^. Chronic dysphagia due to narrowing of the neopharyngeal or proximal esophageal lumen rarely occurs due to stricture formation^4^. But chronic dysphagia and chronic voice disorder are also very common after radiochemotherapy for advanced laryngeal and hypopharyngeal cancer^5,6^.

The current knowledge on oncological and functional outcome on locally advanced laryngeal and hypopharyngeal cancer is mainly based on large multicenter prospective phase III clinical trial or on retrospective multicenter cohorts on specialized cancer centers^3,7–9^. The former have the disadvantage of patient selection in a highly standardized setting, are focused on non-surgical treatment, but mostly do not address long-term effects. In contrast, retrospective trials may be the subject to a selection bias. Population-based studies have the advantage to include all cancer patients within a given jurisdiction, are therefore less prone to selection and referral biases. Moreover, they reflect the routine cancer care^10^. The National Cancer Institute’s Surveillance, Epidemiology, and End Results (SEER) registry, the National Cancer Database (NCDB), or national cancer registries record patient and tumor characteristics as well as survival data, but typically not data on functional outcome^11–13^. To overcome this limitation, all eight Department of Otorhinolaryngology, Head and Neck Surgery in Thuringia, a federal state in Germany, have established the Thuringian Head And Neck Cancer Study (THANCS) Group allowing population-based analysis of all head and neck cancer patients treated in one German federal state with about two million inhabitants^14,15^. Furthermore, the network established a linkage between data from all five Thuringian cancer registries and the data of the patients’ charts in the eight hospitals, i.e. a linkage between registry-based and hospital-based data. This allows a linkage to data not recorded in cancer registries^16,17^.

Using the described population-based approach, data of patients with advanced laryngeal and hypopharyngeal cancer were linked to data related to voice and swallowing rehabilitation in all patients who received a laryngectomy between 2001 and 2020 in the entire population of Thuringia. This allowed a comprehensive analysis of the functional and oncological outcomes in a clinical-routine setting beyond clinical trials and specialized cancer centers.

## Methods

### Study design

This population-based, retrospective study included all patients with laryngeal or hypopharyngeal cancer undergoing laryngectomy between 2001 and 2020 (20 years) in Thuringia, Germany. All patients with the following operation and procedure classification system (OPS; version 20) codes were included: 5–303.0 to 5–303.07; 5–303.1 to 5–303.17; 5–303.2 to 5–303.27). As one hospital (Arnstadt) did not perform laryngectomies in this selected time period, this study included all 617 patients from the remaining seven department of otolaryngology in Thuringia (Bad Salzungen, Erfurt, Gera, Jena, Nordhausen, Suhl, Weimar). The patient data set was double-checked against the data sets of the five Thuringian cancer registries (Erfurt, Gera, Jena, Nordhausen, Suhl). So, no patient was overlooked. The patients’ charts were reviewed. All relevant parameters (patient characteristics, tumor-specific data, surgery, aftercare, voice rehabilitation, nutrition, and follow-up) were anonymously recorded in a data base.

All experimental procedures with human subjects followed the institutional research committee's ethical standards and the 1964 Helsinki Declaration and its later amendments. The Ethics Committee of the Jena University Hospital approved the retrospective study of clinical routine data (No. 2018-1075-Material; No. 2022-2526-BO). Informed consent of the patients was waived by the approving ethics committee, as this study had a non-interventional retrospective design and all data were analyzed anonymously.

### Statistical analysis

The statistical analyses were performed with SPSS Statistics version 29.0.0.0 (IBM Deutschland GmbH, 71139 Ehningen, Germany). Nominal and ordinal data are presented as absolute number and percentage. Metric data are presented as mean, standard deviation, median, and range. Time-dependent statistics were needed for the three primary outcome parameters (OS, percutaneous endoscopic gastrostomy [PEG] dependency, maintenance of voice prosthesis use), because of the variable follow-up time of the patients. First, Kaplan–Meier statistics and the log-rank test were used to analyze to impact of variables on OS, duration of the use of a PEG, and maintenance of voice prosthesis use. The significance level was set to p ≤ 0.05. All significant factors from these univariate analyses were included in multivariate analyses using Cox proportional hazard ratio (HR) with corresponding 95% confidence intervals (CI). Furthermore, for the comparisons between patients with laryngeal versus hypopharyngeal cancer, Pearson’s chi-square test or Fisher’s exact test were needed for the nominal/ordinal data. The Mann–Whitney U-test was used to compare metric data. The significance level was again set to p ≤ 0.05.

The epidemiological calculations were performed using the absolute case numbers and data from the Thuringian State Office of Statistics (https://www.statistik.thueringen.de/) on the population of Thuringia during the observation period. The incidences of laryngectomy (surgical rate per 100,000 persons) per year and gender were calculated.

### Ethics statement

The Ethics Committee of the Jena University Hospital approved the retrospective study of clinical routine data (No. 2018-1075-Material; No. 2022-2526-BO). Informed consent of the patients was waived, as this study had a non-interventional retrospective design and all data were analyzed anonymously.

## Results

### Patient’s and tumor characteristics, treatment and complications during long-term follow-up

The characteristics of the patients undergoing laryngectomy for laryngeal or hypopharyngeal cancer in Thuringia in 2001–2020 are shown in Table 1. A total of 617 patients were included in the study. Most patients (93.7%) were male. The median age was 62 years). Three quarters were smokers prior to laryngectomy (77.5%). Four out of five patients were drinking alcohol (82.2). About two third had the primary tumor in the larynx (68.7%) and about one third in the hypopharynx (31.3%). Most frequent tumors were pT4 (45.1%), pN0 (50.2%). A minority had distant metastasis at the time of diagnosis (M+; 2.4%). This resulted in a UICC stage IV in the majority of patients (66.0%).

**Table 1:** Patients’ characteristics and tumor characteristics.

Parameter	Frequency (N)	%
All	617	100
Gender		
Male	578	93.7
Female	39	6.3
Cigarette smoking		
Yes	478	77.5
No	73	11.8
Unknown	66	10.7
Alcohol drinking		
Yes	507	82.2
No	46	7.5
Unknown	64	10.4
Tumor localization		
Larynx	424	68.7
Supraglottic	115	18.6
Glottic	167	27.1
Subglottic	21	3.4
Transglottic	121	19.6
Hypopharynx	193	31.3
pT classification		
T2	80	13.0
T3	248	40.2
T4	278	45.1
TX	11	1.8
pN classification		
N0	310	50.2
N1	60	9.7
N2	219	35.5
N3	17	2.8
NX	11	1.8
M classification		
M0	591	95.8
M1	15	2.4
MX	11	1.8
UICC staging		
Stage II	42	6.8
Stage III	157	25.4
Stage IV	407	66.0
Stage unknown	11	1.8
Grading		
G1	22	3.6
G2	433	70.2
G3	148	24.0
GX	14	2.3

	Mean ± SD	Median, range
Age in years	62.19 ± 9.65	62, 38–89

The treatment characteristics are listed in Table 2. Three quarters received the laryngectomy as primary treatment (75.7%). One quarter was treated for a recurrent tumor (24.3%). Laryngectomy was performed without resection of parts of the pharynx in 57.5%, and with pharyngectomy in 42.5% of the cases. Only a minority of patients needed a reconstruction of the neopharynx with a flap (13.0%). Nearly all patients underwent bilateral neck dissection (96.8%). Three quarters (73.4%) needed a postoperative adjuvant treatment (radiotherapy, radiochemotherapy, or chemotherapy). Chemotherapy as single postoperative therapy was used in some M+ patients. The postoperative complications are summarized in Supplement Table 1. The two most frequent complications were: a pharyngocutaneous fistula (20.4%) and disturbed wound healing (19.0%).

**Table 2:** Treatment characteristics.

Parameter	Frequency (N)	%
All	617	100
First or recurrent tumor treatment		
First tumor treatment	467	75.7
Recurrent tumor treatment	150	24.3
Prior surgery	130	21.1
Prior radiotherapy	81	13.1
Treatment		
Laryngectomy	617	100
Without pharyngectomy	355	57.5
With pharyngectomy	262	42.5
No flap	537	87.0
Regional flap	27	4.4
Distant flap	44	7.1
Microvacular free flap	9	1.5
With thyroid resection	44	7.1
Neck dissection, bilateral	597	96.8
No neck dissection	3	0.5
Neck dissection unknown	17	2.8
Postoperative adjuvant treatment		
No	138	22.4
Radiotherapy	295	47.8
Radiochemotherapy	143	23.2
Chemotherapy	15	2.4
Unknown	26	4.2
Postoperative nutrition		
Nasogastric tube	449	72.8
NGT still in place at last follow-up	2	0.3
Percutaneous endoscopic gastrostomy	146	23.7
PEG in place at last follow-up	52	8.4
Parenteral	5	0.8
Unknown	17	2.8

	Mean ± SD	Median, range
Number of laryngectomies/center/year	4.2** ± **3.7	2.9, 0.1–11.24
Duration of NGT nutrition in days	12.4 ± 9.8	10, 1–103
Duration of PEG nutrition in months	5.8 ± 7.3	3, 0–50

NGT = nasogastric tube; PEG = percutaneous endoscopic gastrostomy.

Nutrition was ensured by a nasogastric feeding tube (NGT; median time of feeding: 10 days) in the majority of the patients (72.8%). 23.7% were fed via a PEG (median time of feeding: 3 months). Data on rehabilitation including speech rehabilitation are listed in Supplement Table 2. 461 patients (74.7%) received a tracheoesophageal puncture and a voice prosthesis for speech rehabilitation. Half of the patients learned esophageal speech (52.7%). Median time of inpatient speech therapy was four days.

Long-term complications are seen in Supplement Table 2. 12.5% of the patient developed a tumor recurrence during the follow-up time. 15.4% developed a secondary primary (mainly lung cancer, 7.8%). 22.9% of the patients died. Chronic or recurrent dysphagia (43.9%) and chronic neck pain (30.1%) were the two most frequent chronic complaints. Table 3 gives an overview about complications related to the voice prosthesis placement and related surgery. The most frequent problems were: leakage through the prosthesis (38.2%), recurrent dysphonia (33.8%), and obstruction of the prosthesis (24.9%). The three most frequent voice related treatments were: dilatation of the esophagus (13.0%), tracheostomy revision (12.6%), and botulinumtoxin injection into the parapharyngeal musculature (10.2%). During follow-up, the median number of voice prosthesis changes was two. The median time to the first change was 7 months. 6.5% of the patients needed definitive removal of the prosthesis and closure of the puncture. The median time to definitive removal in this subgroup of patients was 15.5 months.

**Table 3:** Complications after tracheoesophageal prosthesis placement and related surgery.

Parameter	Frequency (N)	%
All	617	100
Voice prosthesis status		
Patients with voice prosthesis	461	74.7
Patients without voice prosthesis	145	23.5
Patient did not want a prosthesis	57	9.2
Intraoperative contraindication	51	8.3
No suitability after speech diagnostics	17	2.8
Voice prosthesis status unknown	11	1.8
All patients with prosthesis	461	100
Prosthesis related complications		
Leakage through the prosthesis	176	38.2
Recurrent dysphonia	156	33.8
Obstruction of the prosthesis	115	24.9
Dislocation of the prosthesis	44	9.5
Enlarged tracheoesophageal fistula	44	9.5
Infection/granuloma around prosthesis	29	6.3
Extrusion of the prosthesis	16	3.5
Prosthesis complications related therapy		
Dilatation of the esophagus	60	13.0
Tracheostomy revision	58	12.6
Botulinumtoxin injection^a^	47	10.2
Definitive prosthesis removal	30	6.5
Closure of the tracheoesophageal fistula	29	6.3

	Mean ± SD	Median, range
Number of prosthesis changes	3.1 ± 3.3	2, 0–25
Time to first prosthesis change, in months	7.7 ± 5.3	7, 0–22
Time to definitive removal, in months	29.1 ± 32.6	15.5, 0–121
Time to last follow-up with prosthesis in place, in months	36.1 ± 41.8	19, 0–201

^a^In parapharyngeal musculature.

### Overall survival

**Figure 1:**
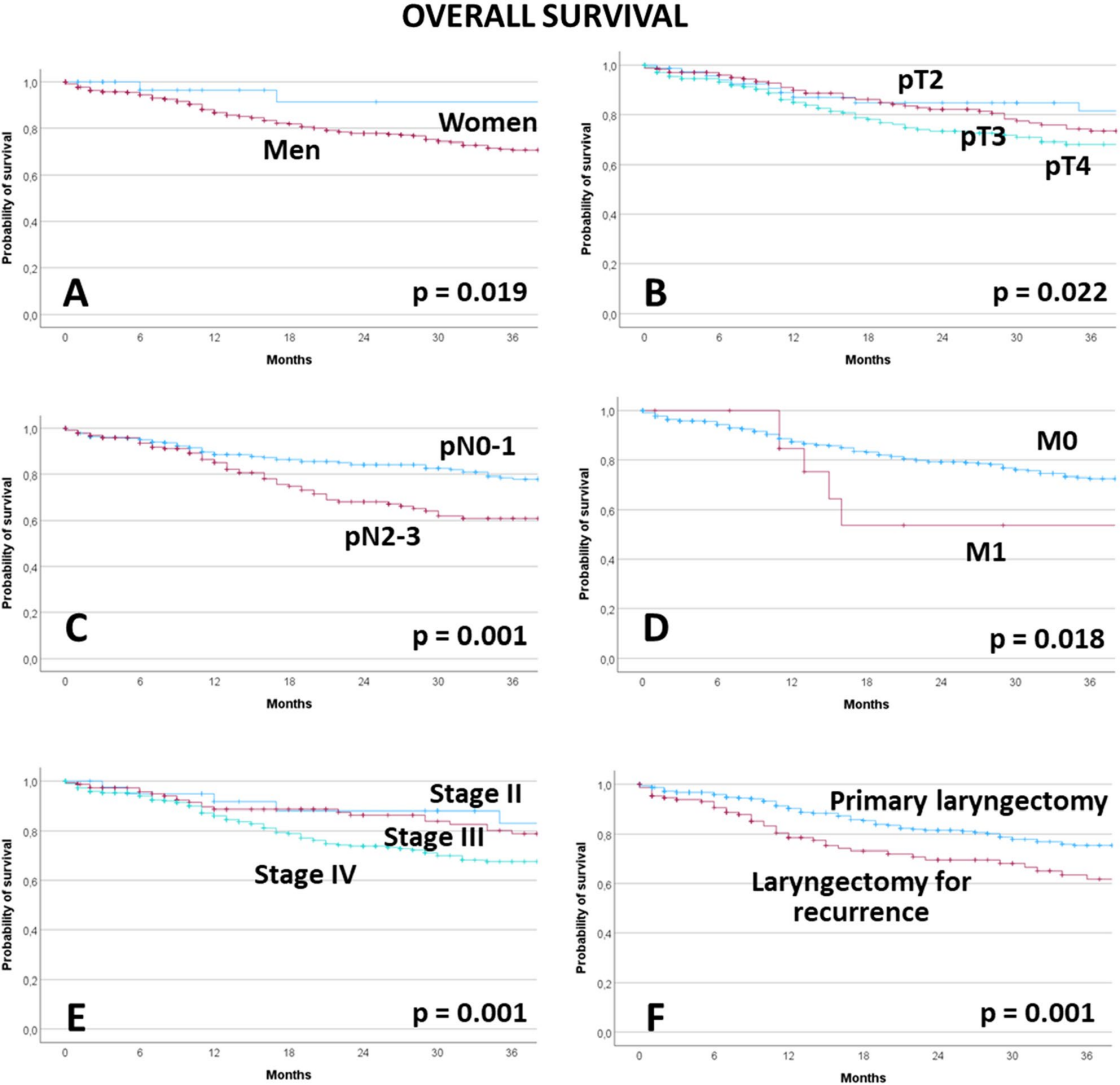
Overall survival, Kaplan–Meier curves and influencing factors. (**A**) Gender. (**B**) pT classification. (**C**) pN classification. (**D**) M classification. (**E**) UICC stage. (**F**) Primary laryngectomy or laryngectomy for tumor recurrence.

The median follow-up time of all patients was 16 months. The median follow-up time of all patients still alive was 17 months. The 5-year OS rate of all patients was 67.2%. The results of the univariate analyses of factors associated to worse OS is presented in Supplement Table 4. Male patients, higher pT classification, higher pN classification, M+, higher UICC stage, and laryngectomy for tumor recurrence were related to worse OS (all p < 0.05; Fig. 1). The multivariate analyses are presented in Table 4. Model 1 included the TNM classification, model 2 the UICC stage. In model 1, pT4 had significantly lower OS than pT2 (HR = 1.947; CI 1.029–3.686; p = 0.041). pN2 had lower OS than pN0 (HR = 1.891; CI 1.304–2.741; p = 0.001). Laryngectomy of a recurrent tumor was associated with lower OS than primary laryngectomy (HR = 1.711; CI 1.167–2.508; p = 0.006). In model 2, stage IV showed lower OS than stage II (HR = 3.455; CI 1.395–8.556; p = 0.007). And again, laryngectomy for a recurrent tumor was followed by lower OS than primary laryngectomy (HR = 1.550; CI 1.078–2.228; p = 0.018).

**Table 4:** Multivariable Cox regression analysis for associations to lower overall survival.

Model 1: Effect of gender, TNM classification, and primary versus recurrent disease treatment			
Parameter	HR	95%-CI	p
Gender			
Female	1	Reference	
Male	3.005	0.953–9.475	0.060
pT classification			
T2	1	Reference	
T3	1.555	0.807–2.995	0.187
T4	1.947	1.029–3.686	**0.041**
pN classification			
N0	1	Reference	
N1	0.949	0.485–1.859	0.879
N2	1.891	1.304–2.741	**0.001**
N3	1.513	0.471–4.866	0.487
M classification			
M0	1	Reference	
M1	2.100	0.966–4.563	0.061
Laryngectomy for			
Primary treatment	1	Reference	
Tumor recurrence	1.711	1.167–2.508	**0.006**

Model 2: Effect of gender, UICC stage, and primary versus recurrent disease treatment			
Gender			
Female	1	Reference	
Male	2.972	0.942–9.372	0.063
UICC staging			
Stage II	1	Reference	
Stage III	2.154	0.827–5.610	0.116
Stage IV	3.455	1.395–8.556	**0.007**
Laryngectomy for			
Primary treatment	1	Reference	
Tumor recurrence	1.550	1.078–2.228	**0.018**

Only significant parameters from the univariate analyses were included in the multivariate analyses. As the UICC stage was derived from the TNM classification, both parameters had to be analyzed in two different models.

Significant values are in bold.

### Duration of nutrition via PEG

**Figure 2:**
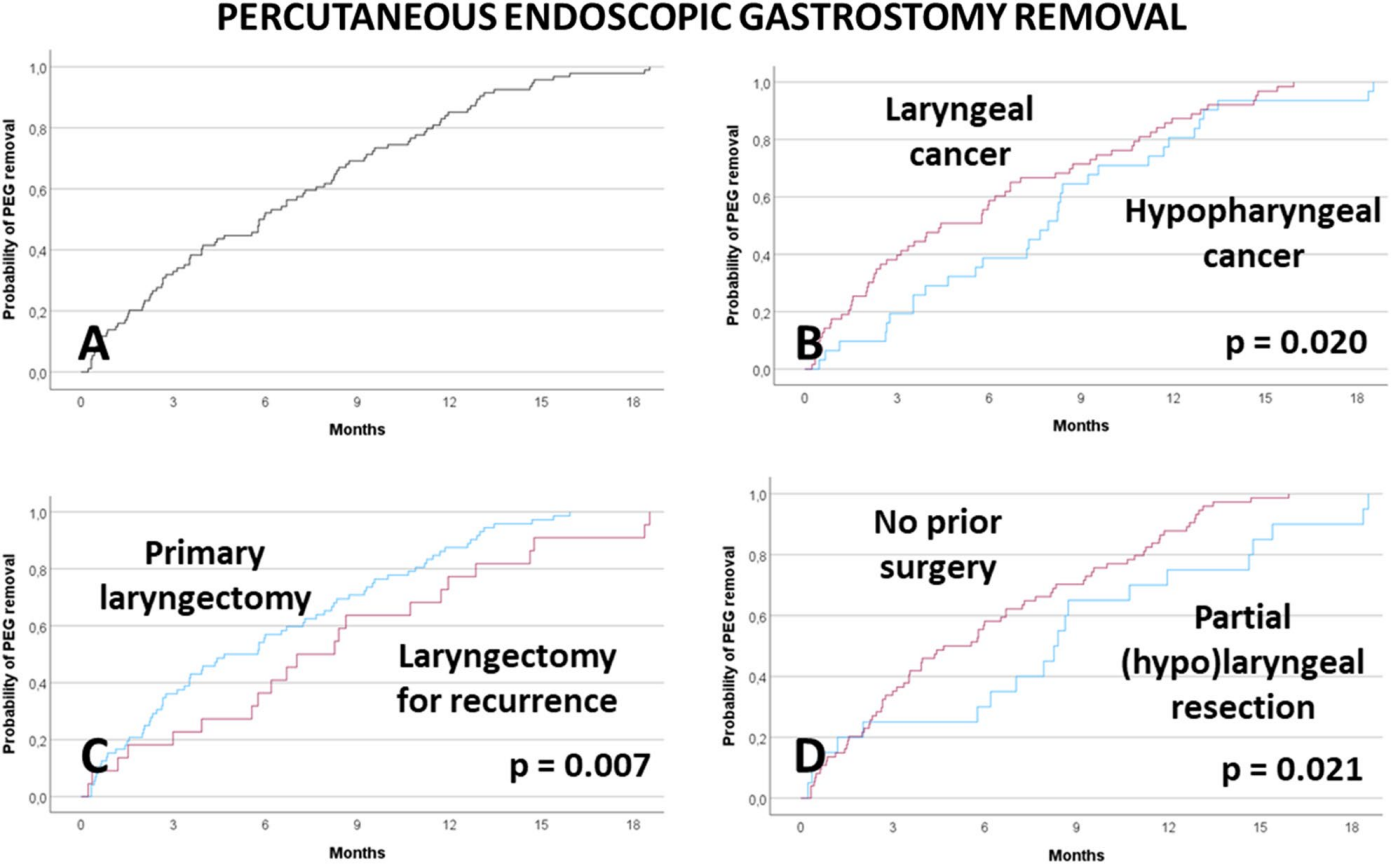
Percutaneous endoscopic gastrostomy (PEG) removal, Kaplan–Meier curves and influencing factors. (**A**) all patients with PEG. (**B**) Primary tumor localization. (**C**) Primary laryngectomy or laryngectomy for tumor recurrence. (**D**) Partial transoral tumor resection prior to laryngectomy (no/yes).

The results of the univariate analyses of factors associated to earlier removal of the PEG and return to oral nutrition over time are shown in Supplement Table 5. The overall 6-month and 12-month PEG removal rates were 42.7% and 84.7%, respectively. None smoking, laryngeal cancer, laryngectomy for primary tumor, and no prior partial resection were the factors associated to earlier removal (all p < 0.05; Fig. 2). The multivariate analyses are presented in Table 5. None of the factors revealed in univariate analysis was an independent factor for earlier removal of the PEG. At most, patients without prior partial surgery before laryngectomy compared to prior surgery showed a tendency for independent association for earlier PEG removal (HR = 1.959; CI 0.921–4.167; p = 0.081).

**Table 5:** Multivariable Cox regression analysis for associations to earlier PEG removal.

Parameter	HR	95%-CI	p
Cigarette smoking			
Yes	1	Reference	
No	0.978	0.517–1.850	0.944
Tumor localization			
Hypopharynx	1	Reference	
Larynx	1.322	0.828–2.113	0.243
Laryngectomy for			
Treatment of tumor recurrence	1	Reference	
Primary treatment	1.126	0.559–2.266	0.740
Prior laryngeal/ hypopharyngeal surgery			
Yes	1	Reference	
No	1.959	0.921–4.167	0.081

Only significant parameters from the univariate analyses were included in the multivariate analysis.

PEG = percutaneous endoscopic gastrostomy.

### Long-term maintenance of the voice prosthesis use

**Figure 3:**
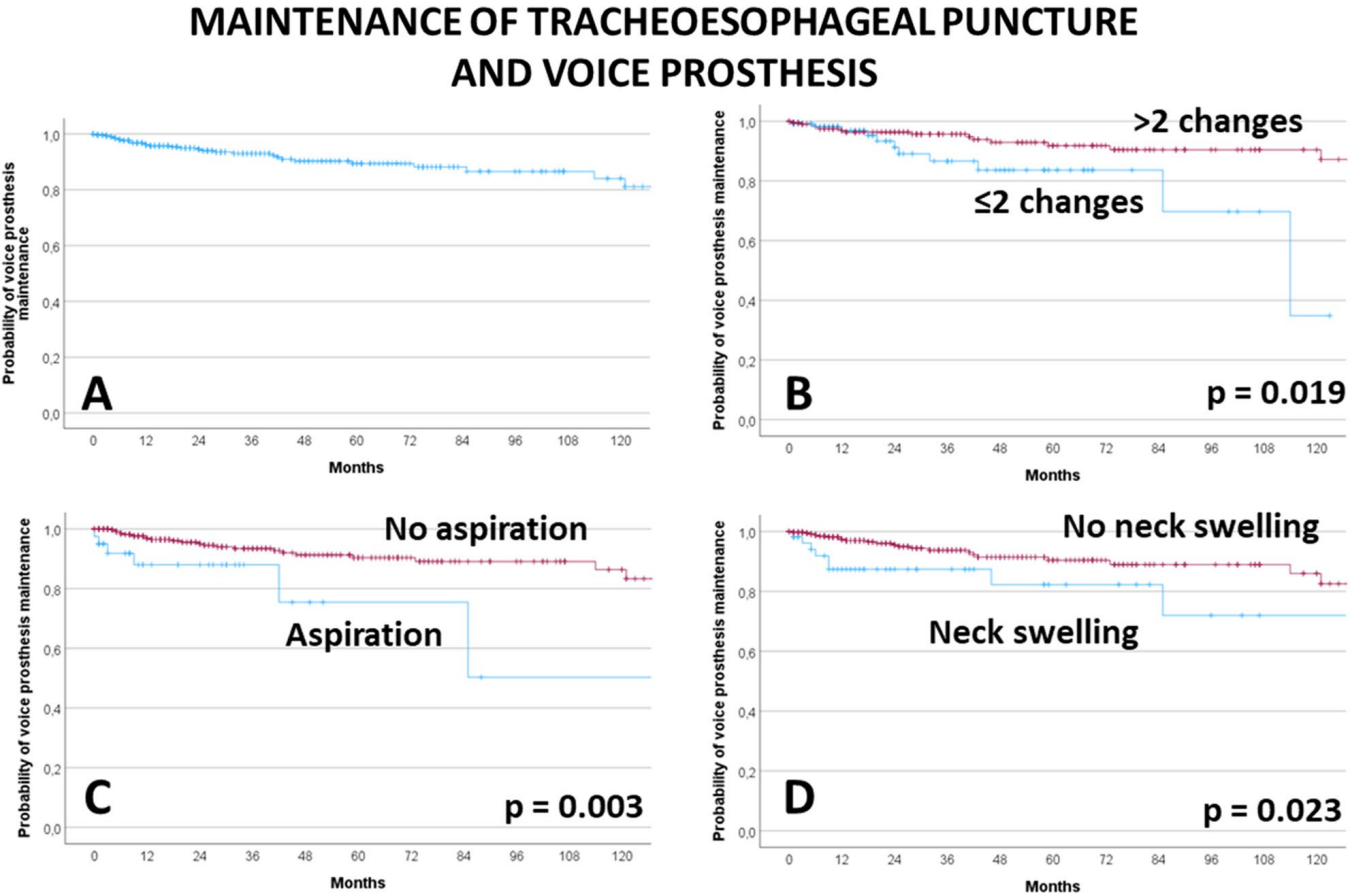
Maintenance of the tracheoesophageal puncture and of the voice prosthesis, Kaplan–Meier curves and influencing factors. (**A**) All patients with voice prosthesis. (**B**) <> median number of voice prosthesis changes (<> 2). (**C**) Postoperative aspiration needing treatment (no/yes). (**D**) Postoperative neck swelling needing treatment (no/yes).

The voice prosthesis was removed during the follow-up in 6.5% of patients. The 5-year definitive voice prosthesis maintenance rate was 89.4%. The results of the univariate analyses of factors associated to long-term use of the voice prosthesis is presented in Supplement Table 6. Adjuvant postoperative chemotherapy, less than the median of two voice prosthesis changes, serious postoperative aspiration needing treatment, severe wound healing disorders after laryngectomy, and therapy-relevant neck swelling after surgery resulted in a higher risk for definitive voice prosthesis removal during follow-up (all p < 0.05; Fig. 3). The results of the multivariate analyses are presented in Table 6. Three models were calculated. The first model included all significant factors from the univariate analysis. Model 2 omitted the factor number of voice prosthesis changes as permanent removal might be directly related to a lower number of changes. Model 3 also omitted the factor postoperative adjuvant treatment as only 2.4% of the patients received a postoperative chemotherapy as monotherapy. In model 1, only less changes than the median of 2 was associated to higher risk of definitive removal (HR = 2.481; CI 1.063–5.786; p = 0.035). In model 2, postoperative aspiration needing treatment was an independent risk factor (HR = 2.679; CI 1.001–7.167; p = 0.050). The same result was seen in model 3 (HR = 3.126; CI 1.207–8.095; p = 0.019).

**Table 6:** Multivariable Cox regression analysis for associations to definitive voice prosthesis removal*

Parameter	HR	95%-CI	p
Model 1: inclusion of all significant factors from the univariate analysis			
Postoperative adjuvant treatment			
No	1	Reference	
Radiotherapy	1.140	0.372–3.489	0.819
Radiochemotherapy	0.903	0.239–3.406	0.880
Chemotherapy	2.631	0.275–25.165	0.401
Number of prosthesis changes			
> Median 2 changes	1	Reference	
< Median 2 changes	2.481	1.063–5.786	**0.035**
Postoperative aspiration			
No	1	Reference	
Yes	2.524	0.839–7.592	0.099
Disturbed wound healing			
No	1	Reference	
Yes	1.461	0.561–3.800	0.437
Head neck swelling, needing treatment			
No	1	Reference	
Yes	1.871	0.714–4.904	0.202

Model 2: like model 1 but omitting the number of voice prosthesis changes as permanent removal might be directly related to a lower number of changes			
Postoperative adjuvant treatment			
No	1	Reference	
Radiotherapy	1.031	0.374–2.837	0.954
Radiochemotherapy	0.813	0.247–2.677	0.734
Chemotherapy	3.947	0.892–17,464	0.070
Postoperative aspiration			
No	1	Reference	
Yes	2.679	1.001–7.167	**0.050**
Disturbed wound healing			
No	1		
Yes	1.398	0.598–3.268	0.439
Head neck swelling, needing treatment			
No	1	Reference	
Yes	2.218	0.967–5.087	0.060

Model 3: like model 2 but omitting the factor postoperative adjuvant treatment as only 2.4% of the patients received a postoperative chemotherapy as monotherapy			
Postoperative aspiration			
No	1	Reference	
Yes	3.126	1.207–8.095	**0.019**
Disturbed wound healing			
No	1	Reference	
Yes	1.480	0.632–3.468	0.366
Head neck swelling, needing treatment			
No	1	Reference	
Yes	2.268	0.985–5.227	0.054

Only significant parameters from the univariate analyses were included in the multivariate analyses.

Significant values are in bold.

### Incidence of laryngectomy between 2001 and 2020

**Figure 4:**
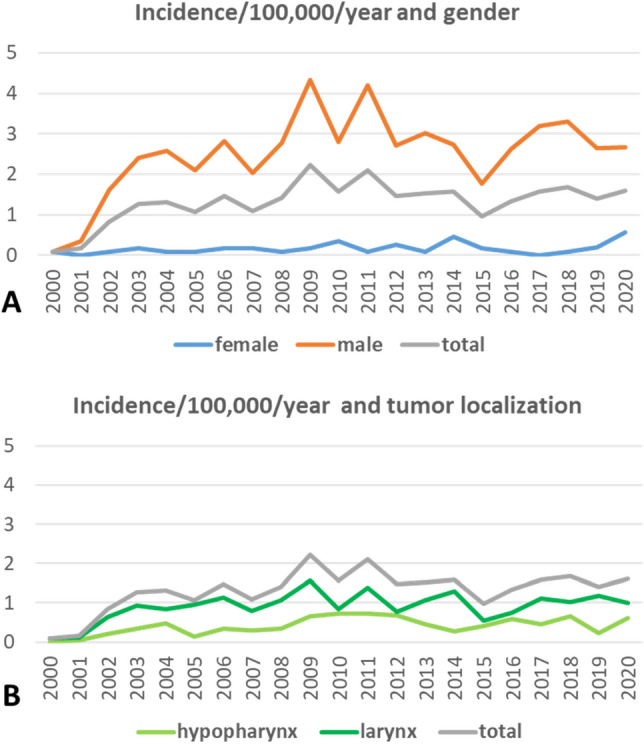
Incidence (surgical rate) of laryngectomy in Thuringia from 2001 to 2020. (**A**) Related to gender. (**B**) Related to the primary tumor localization.

The Thuringian population varied between 2001 and 2020 between 2,120,237 and 2,431,255 people (female: 1,071,025–1,241,304; male: 1,049,212–1,189,951). The average incidence of laryngectomy in the observed 20 years was 1.32 ± 0.51 (Supplement Table 7). The laryngectomy rate was considerably higher in men (2.51 ± 1.01) than in women (0.17 ± 0.14). The laryngectomy rate was double as high in patients with advanced laryngeal cancer (1.85 ± 4.17) than in patients with hypopharyngeal cancer (0.85 ± 1.90). The incidence rate varied over the years in both genders and tumor locations, but a decrease over time was not seen (Fig. 4).

### Comparison of patients with laryngeal cancer versus patients with hypopharyngeal cancer

A comparison of the two laryngectomy groups was not the primary objective of the present study. Explorative data on the comparisons between patients with laryngeal cancer and hypopharyngeal cancer are summarized in the Supplement Tables 8–14. The groups showed some differences. Briefly, patients with hypopharyngeal cancer had a higher N classification, a higher UICC staging, were younger, had less frequently a prior therapy, and a higher probability for postoperative adjuvant therapy than patients with laryngeal cancer (all p < 0.05). The occurrence of most voice prosthesis-related complications and treatments were not different, except for a lower frequency of tracheostomy revisions (p = 0.019) and need for botulinumtoxin injection (p = 0.025) in patients with hypopharyngeal cancer. Postoperative bleeding was more frequent after laryngectomy for hypopharyngeal cancer (p = 0.026). Some long-term complications were more frequent in patients with laryngeal cancer: more chronic/recurrent dysphagia (p = 0.020), more frequently tracheostomy dysfunction (p = 0.018), but less frequent chronic neck pain (p = 0.008).

## Discussion

Laryngectomy with neck dissection followed by post-operative radiotherapy or radiochemotherapy is a standard treatment option for patients with locally advanced laryngeal or hypopharyngeal cancer. Despite the establishment of organ preservation strategies by radiochemotherapy, the use of laryngectomy is not declining^18,19^. Nevertheless, epidemiology studies of the general trend of advanced laryngeal and hypopharyngeal cancer undergoing laryngectomy in a population-based setting are sparse. The present study provides important population-based and therefore real world setting insights into the oncological and at the same time functional outcome after laryngectomy. Concerning the functional evaluation, we are not aware of any other comparable population-based study.

The incidence numbers over time showed that laryngectomy is still and consistently an important treatment option for advanced laryngeal and hypopharyngeal cancer in Germany. When laryngectomy is compared to non-surgical approaches in population-based studies, often only relative numbers in percentage are reported^18–20^. At least, the use of laryngectomy for advanced laryngeal cancer seems not to decrease in the United States^19,20^. In contrast, a relative decrease of the use of laryngectomy is reported for hypopharyngeal cancer in the Netherlands^12^.

The 5-year OS of all patients was 67.2%. This is comparable to results of other population-based studies on laryngectomy ± postoperative radio(chemo)therapy^12,13,19–22^, or even better than in some populations^23^. Of course, such comparisons of the results of different studies should be viewed with caution, because a relevant selection bias cannot be excluded. Anyhow, in accordance to other studies, higher tumor staging and treatment for a recurrent tumor were to most predictive factor for lower OS also in the present study^19,20,24^.

Prospective trials of post-laryngectomy complications are sparse^18,25^. This makes the population-based approach even more meaningful. The rates for the two most frequent complications, i.e. pharyngocutaneous fistula (20.4%) and disturbed wound healing (19.0%) were not higher than in other retrospective hospital-based studies^2,26^. Laryngectomy and postoperative radiotherapy have several effects on swallowing function because of the anatomic changes due to the neopharynx construction, and the intrapharyngeal pressure changes^27^. The altered swallowing dynamics and stenosis are the most important causes of dysphagia^27,28^. Post-laryngectomy dysphagia rates vary from 10 to 62%^27^. As it is standard, most patients (72.8%) in the present study only needed about 10 days of nutrition by a nasogastric tube to overcome the early postoperative phase. Nevertheless, about a quarter (23.7%) needed a PEG associated with a 12-month PEG removal rates of 84.7%. This rate was also seen in a recent population-based study from the United States^18^. Here, 14% and 7%, respectively, of patients who received a radiochemotherapy or a total laryngectomy were still PEG dependent after 12 months. Hence, the gastrostomy tube dependence seems to be lower than after radiochemotherapy for this type of advanced cancer^18,29^. Furthermore, chronic or recurrent dysphagia was reported by 36.8% of the patients, i.e. this was not only an issue for the patients needing a PEG. Whereas the univariate analysis showed some factors with influence on earlier removal (no smoking, primary tumor treatment, no prior partial resection), the multivariate analysis did not reveal an independent factor. Hence, longer dependency on the PEG seems to be multifactorial. Even, if not needed anymore, it could take time until the PEG is removed.

Tracheoesophageal speech through a tracheoesophageal fistula and voice prosthesis is the best speech rehabilitation method according to acoustic and perceptual outcomes^30^. We could not identify any population-based study on this topic. The voice prosthesis implantation rate was 74.7%. This number seems to be high but other population-based studies addressing this issue are missing. The rate is considerably higher than reported for the United States^31^. The clear majority received a primary TEP (97%). This is in contrast to other larger series, demonstrating a much more frequent use of secondary TEP^31,32^. Prospective trials typically investigate a new type of voice prosthesis^33^. Despite advances in voice prosthesis design, magnetized valves, and antimicrobial materials, device complications still are common^28^. The prostheses last only a limited period of time, and require repeated replacements on average every 2–3 months^34^. The average life time of the first voice prosthesis in the present study was 7 months. This is long compared to other studies^32,35,36^, and speaks for a good instruction of the laryngectomees how to handle the prosthesis^28^. Leakage through the prosthesis (38.2%) followed by obstruction of the prosthesis (24.9%) were the most frequent indications for replacement like showed also in other studies^28,37,38^. Data on long term use and maintenance of the TEP are sparse and we were not aware of any population-based study. The 5-year definitive voice prosthesis maintenance rate was 89.4%. During follow-up, the TEP was definitively closed in 6.5% of the patients. This seems to be in the range of the data of specialized centers^37^.

The present study was limited by its retrospective character. The follow-up evaluation was dependent on outpatient visits of the patients in the hospital. Voice prosthesis might have been changed elsewhere, too. Therefore, we probably underestimated the number of prosthesis changes (which should be taken into account, when interpreting the multivariate model 1 in Table 6). Hence, an analysis of the causal relationship between the detected predictive factors and outcome was not possible. Furthermore, several factors that might have influence on oncological and functional outcome, for instance like comorbidity, body mass index and socioeconomic factors, were not analyzed but would be of interest for future studies^39–41^. To directly compare laryngectomy ± postoperative radio(chemo)therapy to non-surgical treatment concepts is difficult in a retrospective population-based setting due to the selection bias^19,20^, but is planned as a next study in the Thuringian setting.

## Conclusions

This retrospective, population-based study describes the oncological and the functional outcome of 617 patients with laryngeal or hypopharyngeal cancer undergoing total laryngectomy in Thuringia between 2001 and 2020. The study comprises a non-selected federal state-wide setting over a period of 20 years, and provides a comprehensive presentation of the trends in incidence, overall survival, and function of laryngeal or hypopharyngeal cancer undergoing laryngectomy in Germany. The surgical rates remained stable over the last 10 years reflecting the unchanged importance of the procedure. Speech rehabilitation with voice prosthesis is standard of care for most patients with a very high success rate. Transient nutrition via percutaneous endoscopic gastrostomy is important for about a quarter of the patients, but removal and return to oral nutrition can be seen within a few months after surgery.

### Supplementary Information

Supplementary Information.

## Data Availability

The original contributions presented in the study are included in the article and in the supplementary material. Further inquiries can be directed to the corresponding author.
