# Supplementary material for: Oncological and functional outcome after laryngectomy for laryngeal and hypopharyngeal cancer: a population-based analysis in Germany from 2001 to 2020
Source: Sci Rep. 2024 Apr 2;14:7761. doi: 10.1038/s41598-024-58423-x (PMC10987613; doi:10.1038/s41598-024-58423-x)
Supplement: Supplementary file 1 — Supplementary Information. [file 41598_2024_58423_MOESM1_ESM.docx]

**Oncological and functional outcome after laryngectomy for laryngeal and hypopharyngeal cancer: a population-based analysis in Germany from 2001 to 2020**

Mussab Kouka^a^, Louise Beckmann^a^, Thomas Bitter^a^, Holger Kaftan^b^, Daniel Böger^c^, Jens Büntzel^d^, Andreas Müller^e^, Kerstin Hoffmann^f^, Jiri Podzimek^g^, Klaus Pietschmann^h^, Thomas Ernst^i^, Orlando Guntinas-Lichius^a,*^

**Complete data set: Supplement Tables 1-7**

**Separately for** **laryngeal and hypopharyngeal cancer: Supplement Tables 8-14**

**Supplement Table 1**

| **Supplement Table 1.** Postoperative complications | | |
| --- | --- | --- |
| **Parameter** | **Frequency (N)** | **%** |
| All | 617 | 100 |
| Bleeding, needing treatment |  |  |
| No | 558 | 90.4 |
| Yes | 59 | 9.6 |
| Aspiration, needing treatment |  |  |
| No | 558 | 90.4 |
| Yes | 59 | 9.6 |
| Pharyngocutaneous fistula |  |  |
| No | 491 | 79.6 |
| Yes | 126 | 20.4 |
| Disturbed wound healing |  |  |
| No | 500 | 81.0 |
| Yes | 117 | 19.0 |
| Head neck swelling, needing treatment |  |  |
| No | 531 | 86.1 |
| Yes | 86 | 13.9 |

**Supplement Table 2**

| **Supplement Table 2.** General rehabilitation and speech rehabilitation | | |
| --- | --- | --- |
| **Parameter** | **Frequency (N)** | **%** |
| All | 617 | 100 |
| Rehabilitation clinic after therapy | 222 | 36.0 |
| Psychooncology support | 108 | 17.5 |
| Postoperative speech therapy |  |  |
| Yes | 521 | 84.4 |
| No | 64 | 10.4 |
| Unknown | 32 | 5.2 |
| Tracheoesophageal speech |  |  |
| Primary prosthesis placement | 451 | 73.1 |
| Secondary prosthesis placement | 10 | 1.6 |
| No prosthesis | 145 | 23.5 |
| Unknown | 11 | 1.8 |
| Esophageal speech |  |  |
| Yes | 325 | 52.7 |
| No | 170 | 27.6 |
| Unknown | 122 | 19.8 |
| Electrolarynx |  |  |
| Yes | 204 | 33.1 |
| No | 381 | 61.8 |
| Unknown | 32 | 5.2 |
| Employability |  |  |
| Return to work | 113 | 18.3 |
| Unable to work | 120 | 19.4 |
| Retired | 225 | 36.5 |
| Unknown | 159 | 25.8 |
|  | **Mean±SD** | **Median, Range** |
| Duration, speech therapy in days | 8.9**±**11.0 | 4, 2-60 |

**Supplement Table 3**

| **Supplement Table 3.** Long-term complications during follow-up | | |
| --- | --- | --- |
| **Parameter** | **Frequency (N)** | **%** |
| **Long-term complication** |  |  |
| All | 617 | 100 |
| Tumor recurrence | 77 | 12.5 |
| Second primary cancer | 95 | 15.4 |
| Lung | 48 | 7.8 |
| Esophagus | 17 | 2.8 |
| Tongue, anterior | 16 | 2.6 |
| Bladder | 8 | 1.3 |
| Skin | 3 | 0.5 |
| Tonsil | 2 | 0.3 |
| Breast | 1 | 0.2 |
| Death | 141 | 22.9 |
| Chronic or recurrent dysphagia | 271 | 43.9 |
| Chronic neck pain | 186 | 30.1 |
| Tracheostomy dysfunction | 138 | 22.4 |
| Severe dyspnea, need to visit doctor | 96 | 15.6 |
| Chronic neck lymphedema | 129 | 20.9 |
| Recurrent tracheitis | 107 | 17.3 |
|  | **Mean±SD** | **Median, Range** |
| Follow-up, all patients, in months | 31.6±39.0 | 16, 0-201 |
| Follow-up, patients alive, in months | 32.6±40.6 | 17, 0-201 |

**Supplement Table 4**

| **Supplement Table 4.** Univariate analysis of associations on overall survival. | | | |
| --- | --- | --- | --- |
| **Parameter** | **5-year overall survival rate in %** | **Log-rank**  **p** |  |
| All | 67.2 |  |  |
| **Patient and tumor characteristics** | | | |
| Gender |  | **0.019** |  |
| Male | 65.4 |  |  |
| Female | 91.4 |  |  |
| Age |  | 0.453 |  |
| <Median 62 years | 67.2 |  |  |
| >Median 62 years | 65.8 |  |  |
| Cigarette smoking |  | 0.798 |  |
| Yes | 66.6 |  |  |
| No | 69.1 |  |  |
| Alcohol drinking |  | 0.984 |  |
| Yes | 67.7 |  |  |
| No | 61.8 |  |  |
| Localization |  | 0.080 |  |
| Larynx | 68.5 |  |  |
| Hypopharynx | 62.2 |  |  |
| pT classification |  | **0.022** |  |
| T2 | 81.5 |  |  |
| T3 | 68.0 |  |  |
| T4 | 60.9 |  |  |
| pN classification |  | **0.013** |  |
| N0 | 70.5 |  |  |
| N1 | 82.4 |  |  |
| N2 | 56.0 |  |  |
| N3 | 64.3 |  |  |
| M classification |  | **0.018** |  |
| M0 | 67.4 |  |  |
| M1 | 26.9 |  |  |
| UICC Staging |  | **0.002** |  |
| Stage II | 83.1 |  |  |
| Stage III | 73.9 |  |  |
| Stage IV | 61.1 |  |  |
| Grading |  | 0.068 |  |
| G1 | 51.7 |  |  |
| G2 | 69.4 |  |  |
| G3 | 61.4 |  |  |
| Laryngectomy for |  | **0.022** |  |
| primary treatment | 69.5 |  |  |
| Treatment of tumor recurrence | 57.6 |  |  |
| **Therapy characteristics** | | | |
| Prior laryngeal/hypopharyngeal surgery |  | 0.873 |  |
| Yes | 68.5 |  |  |
| No | 66.1 |  |  |
| Prior radiotherapy |  | 0.098 |  |
| Yes | 50.7 |  |  |
| No | 69.0 |  |  |
| Laryngectomy |  | 0.735 |  |
| without pharyngectomy | 67.3 |  |  |
| with pharyngectomy | 65.5 |  |  |
| Postoperative adjuvant treatment |  | 0.057 |  |
| No | 59.7 |  |  |
| Radiotherapy | 68.4 |  |  |
| Radiochemotherapy | 73.3 |  |  |
| Chemotherapy | 31.0 |  |  |
| Postoperative nutrition |  | 0.071 |  |
| Nasogastric tube | 69.7 |  |  |
| PEG | 50.8 |  |  |
| Number of laryngectomies/center |  | 0.740 |  |
| ≤median of 2.9 per year | 71.7 |  |  |
| >median of 2.9 per year | 65.7 |  |  |
| Duration of nasogastric tube nutrition |  | 0.571 |  |
| ≤median of 10 days | 69.0 |  |  |
| >median of 10 days | 70.8 |  |  |
| Duration of PEG nutrition |  | 0.746 |  |
| <median of 3 months | 59.3 |  |  |
| ≥median of 3 months | 54.6 |  |  |
| **Postoperative complications** | | | |
| Bleeding, needing treatment |  | 0.123 |  |
| No | 67.5 |  |  |
| Yes | 59.1 |  |  |
| Aspiration, needing treatment |  | 0.105 |  |
| No | 68.1 |  |  |
| Yes | 46.3 |  |  |
| Pharyngocutaneous fistula |  | 0.251 |  |
| No | 68.0 |  |  |
| Yes | 60.2 |  |  |
| Disturbed wound healing |  | 0.449 |  |
| No | 67.6 |  |  |
| Yes | 61.9 |  |  |
| Head neck swelling, needing treatment |  | 0.806 |  |
| No | 66.0 |  |  |
| Yes | 70.5 |  |  |

PEG = percutaneous endoscopic gastrostomy

**Supplement Table 5**

| **Supplement Table 5.** Univariate analysis of associations on PEG dependency. | | | |
| --- | --- | --- | --- |
| **Parameter** | **6-month PEG removal rate in %** | **Log-rank**  **p** |  |
| All | 42.7 |  |  |
| **Patient and tumor characteristics** | | | |
| Gender |  | 0.774 |  |
| Male | 43.4 |  |  |
| Female | 30.0 |  |  |
| Age |  | .0051 |  |
| <Median 62 years | 32.5 |  |  |
| >Median 62 years | 55.1 |  |  |
| Cigarette smoking |  | **0.048** |  |
| Yes | 35.5 |  |  |
| No | 74.2 |  |  |
| Alcohol drinking |  | 0.647 |  |
| Yes | 38.8 |  |  |
| No | 65.7 |  |  |
| Tumor localization |  | **0.020** |  |
| Larynx | 50.8 |  |  |
| Hypopharynx | 29.3 |  |  |
| UICC Staging |  | 0.938 |  |
| Stage II | 50.0 |  |  |
| Stage III | 35.7 |  |  |
| Stage IV | 43.2 |  |  |
| Laryngectomy for |  | **0.007** |  |
| primary treatment | 48.8 |  |  |
| Treatment of tumor recurrence | 26.0 |  |  |
| **Therapy characteristics** | | | |
| Prior laryngeal/hypopharyngeal surgery |  | **0.014** |  |
| Yes | 20.9 |  |  |
| No | 49.6 |  |  |
| Prior radiotherapy |  | 0.389 |  |
| Yes | 31.1 |  |  |
| No | 45.5 |  |  |
| Laryngectomy |  | 0.196 |  |
| without pharyngectomy | 54.4 |  |  |
| with pharyngectomy | 38.6 |  |  |
| Postoperative adjuvant treatment |  | 0.474 |  |
| No | 37.4 |  |  |
| Radiotherapy | 49.5 |  |  |
| Radiochemotherapy | 47.2 |  |  |
| Chemotherapy | 33.7 |  |  |
| Number of laryngectomies/center |  | 0.404 |  |
| ≤median of 2.9 per year | 58.8 |  |  |
| >median of 2.9 per year | 36.5 |  |  |
| **Postoperative complications** | | | |
| Bleeding, needing treatment |  | 0.227 |  |
| No | 46.1 |  |  |
| Yes | 48.5 |  |  |
| Aspiration, needing treatment |  | 0.478 |  |
| No | 43.9 |  |  |
| Yes | 59.2 |  |  |
| Pharyngocutaneous fistula |  | 0.093 |  |
| No | 62.6 |  |  |
| Yes | 30.8 |  |  |
| Disturbed wound healing |  | 0.064 |  |
| No | 50.1 |  |  |
| Yes | 34.8 |  |  |
| Head neck swelling, needing treatment |  | 0.596 |  |
| No | 56.3 |  |  |
| Yes | 49.5 |  |  |

PEG = percutaneous endoscopic gastrostomy

**Supplement Table 6**

| **Supplement Table 6.** Univariate analysis of associations on voice prosthesis maintenance | | | |
| --- | --- | --- | --- |
| **Parameter** | **5-year definitive voice prosthesis maintenance rate in %** | **Log-rank**  **p** |  |
| All | 89.4 |  |  |
| **Patient and tumor characteristics** | | | |
| Gender |  | 0.697 |  |
| Male | 89.7 |  |  |
| Female | 93.0 |  |  |
| Age |  | 0.766 |  |
| <Median 62 years | 88.5 |  |  |
| >Median 62 years | 90.2 |  |  |
| Cigarette smoking |  | 0.919 |  |
| Yes | 88.8 |  |  |
| No | 89.2 |  |  |
| Alcohol drinking |  | 0.753 |  |
| Yes | 88.8 |  |  |
| No | 87.6 |  |  |
| Tumor localization |  | 0.942 |  |
| Larynx | 89.8 |  |  |
| Hypopharynx | 88.6 |  |  |
| UICC Staging |  | 0.269 |  |
| Stage II | 84.4 |  |  |
| Stage III | 88.3 |  |  |
| Stage IV | 90.5 |  |  |
| Laryngectomy for |  | 0.249 |  |
| primary treatment | 90.3 |  |  |
| Treatment of tumor recurrence | 91.4 |  |  |
| **Therapy characteristics** |  |  |  |
| Prior laryngeal/hypopharyngeal surgery |  | 0.134 |  |
| Yes | 83.5 |  |  |
| No | 91.1 |  |  |
| Prior radiotherapy |  | 0.252 |  |
| Yes | 81.8 |  |  |
| No | 90.3 |  |  |
| Laryngectomy |  | 0.406 |  |
| without pharyngectomy | 87.4 |  |  |
| with pharyngectomy | 92.4 |  |  |
| Postoperative adjuvant treatment |  | **0.009** |  |
| No | 87.6 |  |  |
| Radiotherapy | 90.5 |  |  |
| Radiochemotherapy | 91.1 |  |  |
| Chemotherapy | 76.2 |  |  |
| Number of laryngectomies/center |  | 0.378 |  |
| ≤median of 2.9 per year | 89.1 |  |  |
| >median of 2.9 per year | 92.8 |  |  |
| **Prosthesis related complications** |  |  |  |
| Voice prosthesis placement |  | 0.109 |  |
| Primary placement | 89.6 |  |  |
| Secondary placement | 78.8 |  |  |
| Leakage through the prosthesis |  | 0.359 |  |
| Yes | 89.3 |  |  |
| No | 92.3 |  |  |
| Recurrent dysphonia |  | 0.782 |  |
| Yes | 89.4 |  |  |
| No | 91.2 |  |  |
| Obstruction of the prosthesis |  | 0.359 |  |
| Yes | 89.3 |  |  |
| No | 92.3 |  |  |
| Dislocation of the prosthesis |  | 0.581 |  |
| Yes | 89.2 |  |  |
| No | 93.2 |  |  |
| Enlarged tracheoesophageal fistula |  | 0.311 |  |
| Yes | 89.4 |  |  |
| No | 90.3 |  |  |
| Infection/granuloma around prosthesis |  | 0.359 |  |
| Yes | 87.6 |  |  |
| No | 89.6 |  |  |
| Extrusion of the prosthesis |  | 0.077 |  |
| Yes | 81.3 |  |  |
| No | 90.1 |  |  |
| Number of prosthesis changes |  | **0.019** |  |
| <median 2 changes | 83.6 |  |  |
| >median 2 changes | 91.8 |  |  |
| Time to first prosthesis change |  | 0.779 |  |
| <median 7 months | 96.8 |  |  |
| >median 7 months | 90.2 |  |  |
| **Postoperative complications** | | | |
| Bleeding, needing treatment |  |  |  |
| No | 91.8 |  |  |
| Yes | 72.7 |  |  |
| Aspiration, needing treatment |  | **0.003** |  |
| No | 90.4 |  |  |
| Yes | 75.4 |  |  |
| Pharyngocutaneous fistula |  | 0.953 |  |
| No | 89.9 |  |  |
| Yes | 86.7 |  |  |
| Disturbed wound healing |  | **0.042** |  |
| No | 90.5 |  |  |
| Yes | 83.7 |  |  |
| Head neck swelling, needing treatment |  | **0.023** |  |
| No | 90.4 |  |  |
| Yes | 82.3 |  |  |

**Supplement Table 7**

| **Supplement Table 7.** Laryngectomy rates between 2001 and 2020 in Thuringia | | |
| --- | --- | --- |
| **Parameter** | **Mean±SD** | **Median, Range** |
| All patients | 1.32±0.51 | 1.41, 0.08-2.22 |
| Female patients | 0.17±0.14 | 0.09, 0-0.56 |
| Male Patients | 2.51±1.01 | 2.67, 0.08-4.32 |
| Patients with hypopharyngeal cancer | 0.42±0.22 | 0.41, 0-0.73 |
| Patients with laryngeal cancer | 0.91±0.36 | 0.94, 0.08-1.56 |

**Supplement Table 8**

| **Supplement Table 8.** Patients’ characteristics and tumor characteristics | | | | | | | |
| --- | --- | --- | --- | --- | --- | --- | --- |
|  | **All patients** | | **Laryngeal cancer** | | **Hypopharyngeal cancer** | |  |
| **Parameter** | **Frequency (N)** | **%** | **Frequency (N)** | **%** | **Frequency (N)** | **%** | **p** |
| All | 617 | 100 | 424 | 100 | 193 | 100 |  |
| Gender | | |  |  |  |  | 0.943 |
| Male | 578 | 93.7 | 397 | 93.6 | 181 | 93.8 |  |
| Female | 39 | 6.3 | 27 | 6.4 | 12 | 6.2 |  |
| Cigarette smoking | | |  |  |  |  | 0.366 |
| Yes | 478 | 77.5 | 332 | 78.3 | 146 | 75.6 |  |
| No | 73 | 11.8 | 45 | 10.6 | 28 | 14.5 |  |
| Unknown | 66 | 10.7 | 47 | 11.1 | 19 | 9.8 |  |
| Alcohol drinking | | |  |  |  |  | 0.470 |
| Yes | 507 | 82.2 | 343 | 80.9 | 164 | 85.0 |  |
| No | 46 | 7.5 | 34 | 8.0 | 12 | 6.2 |  |
| Unknown | 64 | 10.4 | 47 | 11.1 | 17 | 8.8 |  |
| Tumor localization | | |  |  |  |  |  |
| Larynx | 424 | 68.7 |  |  |  |  |  |
| Supraglottic | 115 | 18.6 |  |  |  |  |  |
| Glottic | 167 | 27.1 |  |  |  |  |  |
| Subglottic | 21 | 3.4 |  |  |  |  |  |
| Transglottic | 121 | 19.6 |  |  |  |  |  |
| Hypopharynx | 193 | 31.3 |  |  |  |  |  |
| pT classification | | |  |  |  |  | 0.825 |
| T2 | 80 | 13.0 | 55 | 13.2 | 25 | 13.0 |  |
| T3 | 248 | 40.2 | 175 | 42.1 | 73 | 37.8 |  |
| T4 | 278 | 45.1 | 186 | 43.9 | 92 | 47.7 |  |
| TX | 11 | 1.8 | 8 | 1.9 | 3 | 1.6 |  |
| pN classification | | |  |  |  |  | **<0.001** |
| N0 | 310 | 50.2 | 258 | 60.8 | 52 | 26.9 |  |
| N1 | 60 | 9.7 | 37 | 8.7 | 23 | 11.9 |  |
| N2 | 219 | 35.5 | 114 | 26.9 | 105 | 54.4 |  |
| N3 | 17 | 2.8 | 7 | 1.7 | 10 | 5.2 |  |
| NX | 11 | 1.8 | 8 | 1.9 | 3 | 1.6 |  |
| M classification | | |  |  |  |  | 0.946 |
| M0 | 591 | 95.8 | 406 | 95.8 | 185 | 95.9 |  |
| M1 | 15 | 2.4 | 10 | 2.4 | 5 | 2.6 |  |
| MX | 11 | 1.8 | 8 | 1.9 | 3 | 1.6 |  |
| UICC Staging | | |  |  |  |  | **<0.001** |
| Stage II | 42 | 6.8 | 37 | 8.7 | 5 | 2.6 |  |
| Stage III | 157 | 25.4 | 122 | 28.8 | 35 | 18.1 |  |
| Stage IV | 407 | 66.0 | 257 | 60.6 | 150 | 77.7 |  |
| Stage unknown | 11 | 1.8 | 8 | 1.9 | 3 | 1.6 |  |
| Grading | | |  |  |  |  | 0.105 |
| G1 | 22 | 3.6 | 20 | 4.7 | 2 | 1.0 |  |
| G2 | 433 | 70.2 | 291 | 68.6 | 142 | 73.6 |  |
| G3 | 148 | 24.0 | 102 | 24.1 | 46 | 23.8 |  |
| GX | 14 | 2.3 | 11 | 2.6 | 3 | 1.6 |  |
|  | **Mean±SD** | **Median, Range** | **Mean±SD** | **Median, Range** | **Mean±SD** | **Median, Range** |  |
| Age in years | 62.19 ± 9.65 | 62, 38-89 | 63.4 ± 9.8 | 63, 38-89 | 59.6 ± 8.8 | 59, 38-83 | **<0.001** |

**Supplement Table 9**

| **Supplement Table 9.** Treatment characteristics | | | | | | | |
| --- | --- | --- | --- | --- | --- | --- | --- |
|  | **All patients** | | **Laryngeal cancer** | | **Hypopharyngeal cancer** | |  |
| **Parameter** | **Frequency (N)** | **%** | **Frequency (N)** | **%** | **Frequency (N)** | **%** | **p** |
| All | 617 | 100 | 424 | 100 | 193 | 100 |  |
| First or recurrent tumor treatment | | | | | | | **0.001** |
| First tumor treatment | 467 | 75.7 | 305 | 71.9 | 162 | 83.9 |  |
| Recurrent tumor treatment | 150 | 24.3 | 119 | 28.1 | 31 | 16.1 |  |
| Prior surgery | 130 | 21.1 | 106 | 25.9 | 24 | 12.6 | **<0.001** |
| Prior radiotherapy | 81 | 13.1 | 58 | 14.1 | 23 | 12.1 | 0.496 |
| Treatment | | | | | | |  |
| Laryngectomy without pharyngectomy | 355 | 57.5 |  |  |  |  |  |
| Laryngectomy with pharyngectomy | 262 | 42.5 |  |  |  |  |  |
| Neck dissection, bilateral | 597 | 96.8 | 408 | 96.2 | 189 | 97.9 | 0.194 |
| No neck dissection | 3 | 0.5 | 1 | 0.2 | 2 | 1.0 |  |
| Neck dissection unknown | 17 | 2.8 | 15 | 3.5 | 2 | 1.0 |  |
| Postoperative adjuvant treatment | | | | | | | **0.017** |
| No | 138 | 22.4 | 103 | 24.3 | 35 | 18.5 |  |
| Radiotherapy | 295 | 47.8 | 91 | 47.2 | 91 | 48.1 |  |
| Radiochemotherapy | 143 | 23.2 | 59 | 30.6 | 59 | 31.2 |  |
| Chemotherapy | 15 | 2.4 | 4 | 2.1 | 4 | 2.1 |  |
| Unknown | 26 | 4.2 | 4 | 2.1 | 22 | 5.2 |  |
| Postoperative nutrition | | | | | | |  |
| Nasogastric tube | 449 | 72.8 |  |  |  |  |  |
| NGT still in place at last follow-up | 2 | 0.3 |  |  |  |  |  |
| Percutaneous endoscopic gastrostomy | 146 | 23.7 |  |  |  |  |  |
| PEG in place at last follow-up | 52 | 8.4 |  |  |  |  |  |
| Parenteral | 5 | 0.8 |  |  |  |  |  |
| Unknown | 17 | 2.8 |  |  |  |  |  |
|  | **Mean±SD** | **Median, Range** | **Mean±SD** | **Median, Range** | **Mean±SD** | **Median, Range** |  |
| Number of laryngectomies /center/year | 4.2**±**3.7 | 2.9, 0.1-11.24 | 2.9±2.5 | 1.9, 0.1-7.6 | 1.4±1.3 | 1.2, 01-3.8 | **<0.001** |
| Duration of NGT nutrition in days | 12.4±9.8 | 10, 1-103 | 11.9±9.0 | 10, 1-103 | 13.1±10.9 | 10, 4-101 | 0.239 |
| Duration of PEG nutrition in months | 5.8±7.3 | 3, 0.2-18.5 | 5.9±4.8 | 4.4, 0.2-15.9 | 7.9±4.8 | 7.9, 0.5-18.5 | 0.062 |

NGT = nasogastric tube; PEG = percutaneous endoscopic gastrostomy

**Supplement Table 10**

| **Supplement Table 10.** Complications after tracheoesophageal prosthesis placement and related surgery | | | | | | |  |
| --- | --- | --- | --- | --- | --- | --- | --- |
|  | **All patients** | | **Laryngeal cancer** | | **Hypopharyngeal cancer** | |  |
| **Parameter** | **Frequency (N)** | **%** | **Frequency (N)** | **%** | **Frequency (N)** | **%** | **p** |
| All | 617 | 100 | 424 | 100 | 193 | 100 |  |
| **Voice prosthesis status** |  |  |  |  |  |  | 0.955 |
| Patients with voice prosthesis | 461 | 74.7 | 316 | 74.5 | 145 | 75.1 |  |
| Patients without voice prosthesis | 145 | 23.5 | 100 | 23.6 | 45 | 23.3 |  |
| Patient did not want a prosthesis | 57 | 9.2 | 39 | 46.4 | 18 | 43.9 | 0.392 |
| Intraoperative contraindication | 51 | 8.3 | 36 | 42.9 | 15 | 36.6 |  |
| No suitability after speech diagnostics | 17 | 2.8 | 9 | 10.7 | 8 | 19.5 |  |
| Voice prosthesis status unknown | 11 | 1.8 | 8 | 1.9 | 3 | 1.6 |  |
|  |  |  |  |  |  |  |  |
|  |  |  |  |  |  |  |  |
| All patients with prosthesis | 461 | 100 |  |  |  |  |  |
| **Prosthesis related complications** |  |  |  |  |  |  |  |
| Leakage through the prosthesis | 176 | 38.2 | 122 | 40.8 | 54 | 37.8 | 0.541 |
| Recurrent dysphonia | 140 | 31.7 | 100 | 33.4 | 40 | 28.0 | 0.247 |
| Obstruction of the prosthesis | 115 | 24.9 |  |  |  |  |  |
| Dislocation of the prosthesis | 44 | 9.5 | 29 | 9.7 | 15 | 10.5 | 0.795 |
| Enlarged tracheoesophageal fistula | 44 | 10.0 | 34 | 11.4 | 10 | 7.0 | 0.150 |
| Infection/granuloma around prosthesis | 29 | 6.3 | 19 | 6.4 | 10 | 7.0 | 0.800 |
| Extrusion of the prosthesis | 16 | 3.5 | 13 | 4.3 | 3 | 2.1 | 0.236 |
| **Prosthesis complications related therapy** |  |  |  |  |  |  |  |
| Dilatation of the esophagus | 61 | 9.9 | 35 | 8.3 | 26 | 13.5 | 0.119 |
| Tracheostomy revision | 58 | 12.6 | 46 | 10.8 | 12 | 6.2 | **0.019** |
| Botulinumtoxin injection* | 41 | 6.6 | 31 | 7.3 | 10 | 5.2 | **0.025** |
| Definitive prosthesis removal | 30 | 6.5 | 22 | 5.2 | 8 | 4.1 | 0.832 |
|  | **Mean±SD** | **Median, Range** | **Mean±SD** | **Median, Range** | **Mean±SD** | **Median, Range** |  |
| Number of prosthesis changes | 3.1±3.3 | 2, 0-25 | 3.3±0.2 | 3, 0-17 | 3.4±0.3 | 2, 0-25 | 0.447 |
| Time to first prosthesis change, in months | 7.7±5.3 | 7, 0-22 | 7.4±0.9 | 7, 0-22 | 7.9±0.5 | 6.5, 0-18 | 0.349 |
| Time to definitive removal, in months | 29.1±32.6 | 15.5, 0-121 | 33.8±7.7 | 21, 0-212 | 16.1±13.2 | 12.5, 1-43 | **0.027** |
| Time to last follow-up with prosthesis in place, in months | 35.6±41.3 | 19, 0-201 | 39.7±2.5 | 23.5, 0-201 | 26.8±2.7 | 15.0, 0-184 | **<0.001** |

*in parapharyngeal musculature

**Supplement Table 11**

| **Supplement Table 11.** Postoperative complications | | | | | | | |
| --- | --- | --- | --- | --- | --- | --- | --- |
|  | **All patients** | | **Laryngeal cancer** | | **Hypopharyngeal cancer** | |  |
| **Parameter** | **Frequency (N)** | **%** | **Frequency (N)** | **%** | **Frequency (N)** | **%** | **p** |
| All | 617 | 100 | 424 | 100 | 193 | 100 |  |
| Bleeding, needing treatment |  |  |  |  |  |  | **0.026** |
| No | 558 | 90.4 | 391 | 92.2 | 167 | 86.5 |  |
| Yes | 59 | 9.6 | 33 | 7.8 | 26 | 13.5 |  |
| Aspiration, needing treatment |  |  |  |  |  |  | 0.452 |
| No | 558 | 90.4 | 386 | 91.0 | 172 | 89.1 |  |
| Yes | 59 | 9.6 | 38 | 9.0 | 21 | 10.9 |  |
| Pharyngocutaneous fistula |  |  |  |  |  |  | 0.102 |
| No | 491 | 79.6 | 345 | 81.4 | 146 | 75.6 |  |
| Yes | 126 | 20.4 | 79 | 18.6 | 47 | 24.4 |  |
| Disturbed wound healing |  |  |  |  |  |  | 0.565 |
| No | 500 | 81.0 | 341 | 80.4 | 159 | 82.4 |  |
| Yes | 117 | 19.0 | 83 | 19.6 | 34 | 17.6 |  |
| Head neck swelling, needing treatment |  |  |  |  |  |  | 0.304 |
| No | 531 | 86.1 | 369 | 87.0 | 162 | 83.9 |  |
| Yes | 86 | 13.9 | 55 | 13.0 | 31 | 16.1 |  |

**Supplement Table 12**

| **Supplement Table 12.** General rehabilitation and speech rehabilitation | | | | | | | |
| --- | --- | --- | --- | --- | --- | --- | --- |
|  | **All patients** | | **Laryngeal cancer** | | **Hypopharyngeal cancer** | |  |
| **Parameter** | **Frequency (N)** | **%** | **Frequency (N)** | **%** | **Frequency (N)** | **%** | **p** |
| All | 617 | 100 | 424 | 100 | 193 | 100 |  |
| Rehabilitation clinic after therapy | 222 | 39.2 | 156 | 40.9 | 66 | 35.5 | 0.211 |
| Psychooncology support | 108 | 19.5 | 79 | 21.3 | 29 | 15.8 | 0.128 |
| Postoperative speech therapy |  |  |  |  |  |  | **0.026** |
| Yes | 521 | 84.4 | 348 | 82.1 | 173 | 89.6 |  |
| No | 64 | 10.4 | 48 | 11.3 | 16 | 8.3 |  |
| Unknown | 32 | 5.2 | 28 | 6.6 | 4 | 2.1 |  |
| Tracheoesophageal speech |  |  |  |  |  |  | 0.992 |
| Primary prosthesis placement | 451 | 73.1 | 309 | 72.9 | 142 | 73.6 |  |
| Secondary prosthesis placement | 10 | 1.6 | 7 | 1.7 | 3 | 1.6 |  |
| No prosthesis | 145 | 23.5 | 100 | 23.6 | 45 | 23.3 |  |
| Unknown | 11 | 1.8 | 8 | 1.9 | 3 | 1.6 |  |
| Esophageal speech |  |  |  |  |  |  |  |
| Yes | 325 | 52.7 | 222 | 52.4 | 103 | 53.4 | 0.881 |
| No | 170 | 27.6 | 115 | 27.1 | 55 | 28.5 |  |
| Unknown | 122 | 19.8 | 87 | 20.5 | 35 | 18.1 |  |
| Electrolarynx |  |  |  |  |  |  | 0.829 |
| Yes | 204 | 33.1 | 138 | 32.5 | 66 | 34.2 |  |
| No | 381 | 61.8 | 265 | 62.5 | 116 | 60.1 |  |
| Unknown | 32 | 5.2 | 21 | 5.0 | 11 | 5.7 |  |
| Employability |  |  |  |  |  |  | 0.079 |
| Return to work | 113 | 18.3 | 73 | 17.2 | 40 | 24.4 |  |
| Unable to work | 120 | 19.4 | 73 | 17.2 | 47 | 20.7 |  |
| Retired | 225 | 36.5 | 163 | 38.4 | 62 | 32.1 |  |
| Unknown | 159 | 25.8 | 115 | 27.1 | 44 | 22.8 |  |
|  | **Mean±SD** | **Median, Range** | **Mean±SD** | **Median, Range** | **Mean±SD** | **Median, Range** |  |
| Duration, speech therapy in days | 8.9**±**11.0 | 4, 2-60 | 8.6**±**11.0 | 4, 2-60 | 9.4**±**11.0 | 4, 2-60 | 0.200 |

**Supplement Table 13**

| **Supplement Table 13.** Long-term complications during follow-up | | | | | | | |
| --- | --- | --- | --- | --- | --- | --- | --- |
| **Long-term complication** | **All patients** | | **Laryngeal cancer** | | **Hypopharyngeal cancer** | |  |
| **Parameter** | **Frequency (N)** | **%** | **Frequency (N)** | **%** | **Frequency (N)** | **%** | **p** |
| All | 617 | 100 | 424 | 100 | 193 | 100 |  |
| Tumor recurrence | 77 | 12.5 | 59 | 13.9 | 18 | 9.3 | 0.071 |
| Second primary cancer | 95 | 15.4 | 57 | 13.4 | 38 | 19.7 | 0.090 |
| Lung | 48 | 7.8 | 28 | 6.6 | 20 | 10.4 |  |
| Esophagus | 17 | 2.8 | 12 | 2.8 | 5 | 2.6 |  |
| Tongue, anterior | 16 | 2.6 | 9 | 2.1 | 7 | 3.6 |  |
| Bladder | 8 | 1.3 | 5 | 1.2 | 3 | 1.6 |  |
| Skin | 3 | 0.5 | 2 | 0.5 | 1 | 0.5 |  |
| Tonsil | 2 | 0.3 | 9 | 2.1 | 7 | 3.6 |  |
| Breast | 1 | 0.2 | 0 | 0.0 | 1 | 0.5 |  |
| Death | 141 | 22.9 | 96 | 22.6 | 45 | 23.3 | 0.853 |
| Chronic or recurrent dysphagia | 271 | 43.9 | 195 | 46.0 | 76 | 39.4 | **0.020** |
| Chronic neck pain | 186 | 30.1 | 111 | 26.2 | 75 | 38.9 | **0.008** |
| Tracheostomy dysfunction | 138 | 22.4 | 104 | 24.5 | 34 | 17.6 | **0.018** |
| Severe dyspnea, need to visit doctor | 96 | 15.6 | 65 | 15.3 | 31 | 16.1 | 0.896 |
| Chronic neck lymphedema | 129 | 20.9 | 93 | 21.9 | 36 | 18.7 | 0.173 |
| Recurrent tracheitis | 107 | 17.3 | 77 | 18.2 | 30 | 15.5 | 0.238 |
|  | **Mean±SD** | **Median, Range** | **Mean±SD** | **Median, Range** | **Mean±SD** | **Median, Range** |  |
| Follow-up, all patients, in months | 31.6±39.0 | 16, 0-201 | 35.2±41.9 | 18, 0-201 | 23.7±30.4 | 12, 0-184 | **<0.001** |
| Follow-up, patients alive, in months | 32.6±40.6 | 17, 0-201 | 36.2±43.5 | 19, 0-201 | 24.6±32.0 | 19, 0-184 | **<0.001** |

**Supplement Table 14**

| **Supplement Table 14.** Laryngectomy rates between 2001 and 2020 in Thuringia | | | | | | | |
| --- | --- | --- | --- | --- | --- | --- | --- |
|  | **All patients** | | **Laryngeal cancer** | | **Hypopharyngeal cancer** | |  |
| **Parameter** | **Mean±SD** | **Median, Range** | **Mean±SD** | **Median, Range** | **Mean±SD** | **Median, Range** |  |
| All patients | 1.32±0.51 | 1.41, 0.08-2.22 | 1.85±4.17 | 0.98, 0.12-0.98 | 0.85±1.90 | 0.46, 0.04-9.10 |  |
| Female patients | 0.17±0.14 | 0.09, 0-0.56 | 0.23±0.54 | 0.09, 0-2.52 | 0.10±0.24 | 0.08, 0-1.12 |  |
| Male Patients | 2.51±1.01 | 2.67, 0.08-4.32 | 3.51±7.89 | 1.81, 0.25-37.84 | 1.61±3.60 | 0.86, 0.08-17.25 |  |

***
